# Association between Renal Function at Admission and COVID-19 in-Hospital Mortality in Southern Italy: Findings from the Prospective Multicenter Italian COVOCA Study

**DOI:** 10.3390/jcm11206121

**Published:** 2022-10-17

**Authors:** Raffaele Galiero, Vittorio Simeon, Giuseppe Loffredo, Alfredo Caturano, Luca Rinaldi, Erica Vetrano, Giulia Medicamento, Maria Alfano, Domenico Beccia, Chiara Brin, Sara Colantuoni, Jessica Di Salvo, Raffaella Epifani, Riccardo Nevola, Raffaele Marfella, Celestino Sardu, Carmine Coppola, Ferdinando Scarano, Paolo Maggi, Cecilia Calabrese, Pellegrino De Lucia Sposito, Carolina Rescigno, Costanza Sbreglia, Fiorentino Fraganza, Roberto Parrella, Annamaria Romano, Giosuele Calabria, Benedetto Polverino, Antonio Pagano, Fabio Giuliano Numis, Carolina Bologna, Mariagrazia Nunziata, Vincenzo Esposito, Nicola Coppola, Nicola Maturo, Rodolfo Nasti, Pierpaolo Di Micco, Alessandro Perrella, Miriam Lettieri, Luigi Elio Adinolfi, Paolo Chiodini, Ferdinando Carlo Sasso, on behalf of COVOCA Study Group

**Affiliations:** 1 Department of Advanced Medical and Surgical Sciences, University of Campania “Luigi Vanvitelli”, Piazza L. Miraglia 2, 80138 Naples, Italy; 2 Medical Statistics Unit, Department of Physical and Mental Health and Preventive Medicine, University of Campania “Luigi Vanvitelli”, Largo Madonna delle Grazie 1, 80138 Naples, Italy; 3 Ospedale Evangelico Betania, Via Argine 604, 80147 Naples, Italy; 4 Hepatology Unit, Internal Medicine, Area Stabiese Hospital, 80053 Naples, Italy; 5 COVID Center “S. Anna e SS. Madonna della Neve” Hospital, 80042 Boscotrecase, Italy; 6 U.O.C. Infectious and Tropical Diseases, S. Anna e S. Sebastiano Hospital, 81100 Caserta, Italy; 7 Pneumologia Vanvitelli Department of Translational Medical Sciences, University of Campania ‘Luigi Vanvitelli’, AORN Ospedali dei Colli, Via Leonardo Bianchi, 80131 Naples, Italy; 8 Covid Center-Maddaloni Hospital, 80124 Maddaloni, Italy; 9 U.O.C. Infectious Diseases and Neurology, Cotugno Hospital, 80131 Naples, Italy; 10 U.O.C. Infectious Diseases of the Elderly, Cotugno Hospital, 80131 Naples, Italy; 11 U.O.C. Anestesia and Intensive Care Unit, Cotugno Hospital, 80131 Naples, Italy; 12 U.O.C. Respiratory Infectious Diseases, Cotugno Hospital, 80131 Naples, Italy; 13 U.O.C. Pneumology, Moscati Hospital, 83100 Avellino, Italy; 14 IXth Division of Infectious Diseases and Interventional Ultrasound, Cotugno Hospital, 80131 Naples, Italy; 15 “Giovanni da Procida” Hospital, 84126 Salerno, Italy; 16 Emergency and Acceptance Unit, “Santa Maria delle Grazie” Hospital, 80078 Pozzuoli, Italy; 17 Internal Medicine Unit, Ospedale Del Mare, 80147 Naples, Italy; 18 U.O.C. Internal Medicine-Moscati Hospital, 83100 Avellino, Italy; 19 IVth Division of Immunodeficiency and Gender Infectious Diseases, Cotugno Hospital, 80131 Naples, Italy; 20 Department of Mental Health and Public Medicine, Centro COVID A.O.U. Vanvitelli, 80131 Naples, Italy; 21 U.O.S.D. Infectious Diseases Emergency and Acceptance, Cotugno Hospital, 80131 Naples, Italy; 22 Emergency Division, A.O.R.N. “Antonio Cardarelli”, Via Antonio Cardarelli 9, 80131 Naples, Italy; 23 Department of Internal Medicine, Fatebenefratelli Hospital of Naples, 80123 Naples, Italy; 24 Task Force COVID-19 Regione Campania, 80131 Napoli, Italy; 25 Division of Cardiovascular Sciences, Faculty of Biology, Medicine and Health, The University of Manchester, 3.31 Core Technology Facility, 46 Grafton Street, Manchester M13 9NT, UK

**Keywords:** COVID-19, chronic kidney diseases, in-hospital mortality

## Abstract

Background. Evidence has shown a close association between COVID-19 infection and renal complications in both individuals with previously normal renal function and those with chronic kidney disease (CKD). Methods. The aim of this study is to evaluate the in-hospital mortality of SARS-CoV-2 patients according to their clinical history of CKD or estimated glomerular filtration rate (eGFR). This is a prospective multicenter observational cohort study which involved adult patients (≥18 years old) who tested positive with SARS-CoV-2 infection and completed their hospitalization in the period between November 2020 and June 2021. Results. 1246 patients were included in the study, with a mean age of 64 years (SD 14.6) and a median duration of hospitalization of 15 days (IQR 9–22 days). Cox’s multivariable regression model revealed that mortality risk was strongly associated with the stage of renal impairment and the Kaplan–Meier survival analysis showed a progressive and statistically significant difference (*p* < 0.0001) in mortality according to the stage of CKD. Conclusion. This study further validates the association between CKD stage at admission and mortality in patients hospitalized for COVID-19. The risk stratification based on eGFR allows clinicians to identify the subjects with the highest risk of intra-hospital mortality despite the duration of hospitalization.

## 1. Introduction

As of August 2022, the coronavirus 2019 disease (COVID-19) pandemic has involved nearly 500 million people worldwide, with almost 6 million deaths [1]. Although COVID-19 infections still represent a healthcare problem worldwide, the mortality rate, hospitalization, and transmission risk caused by this disease have been reduced due to the spread of COVID-19 vaccines, improved treatments, and increased social awareness. [1,2,3,4]. This global trend has also been confirmed in Italy, where a reduction in the number of hospitalized subjects in intensive care units and mortality rate have been observed [5,6]. Despite this, the daily incident rate of newly infected subjects during the so-called third and fourth waves has still been high, as has the number of patients admitted to medical care units [1,7].

The COVID-19 illness is a multisystem disease, and the complications during and after viral infection are still among the most concerning elements of the pandemic and remain incompletely understood. COVID-19 is typically associated with endothelial inflammation and microvascular thrombosis, which mainly involve the vasculature of the lung, brain, skin, and fat tissues [8]. Angiotensin-converting enzyme 2 (ACE-2) receptors are highly expressed in the aforementioned tissues, thus suggesting its possible role in the microvascular injury. Moreover, a possible role of complement activation in microvascular injury development has been reported in both mouse and human models [9,10,11]. However, the role of ACE-2 receptors on the immune system linked to viral infection is not completely understood and is still debated. 

In a previous article, we described the link between patients having a history of chronic liver disease (CLD) and in-hospital mortality during the first wave of the COVID-19 pandemic in the Campania region of Italy [12]. There is strong evidence suggesting a close association between COVID-19 infection and renal complications in both individuals with normal renal function and chronic kidney disease (CKD) patients. Moreover, other authors have demonstrated that this occurrence can significantly impact a patient’s prognosis [13].

The aim of this study is to evaluate the in-hospital mortality of SARS-CoV-2 patients according to their history of CKD and estimated glomerular filtration rate (eGFR). 

## 2. Materials and Methods

### 2.1. Study Design and Participants

COVOCA (an observational study on the COVID-19 population hospitalized in the Campania region) is a prospective observational cohort study which involved 23 COVID hospital units in the Campania region of Italy. 

We enrolled adult patients (≥18 years old) who tested positive with SARS-CoV-2 infection and completed their hospitalization (discharged or dead) in the period between November 2020 and June 2021. Patients with either missing or incomplete laboratory and clinical data at hospitalization and at the time of discharged were excluded from the study. 

Clinical charts and hospital electronic records were used as sources of data. The study was approved by the local ethics committees and was in accordance with the 1976 Declaration of Helsinki and its later amendments. 

### 2.2. Variables (Outcome and Exposure)

The microbiological diagnosis of SARS-CoV-2 infection was defined by the real-time polymerase chain reaction of nasal-pharyngeal swab specimens. 

Data on in-hospital mortality and in-hospital length of stay were obtained from either death certificates or discharge letters. The exposure variables were collected during hospital admission as follows: (a) demographic and anthropometric characteristics (sex, age, and real-time PCR swab specimen); (b) anamnestic data (number of COVID-19 positive cases in the family, number of vaccinated subjects, type of vaccine, and days passed from diagnosis to hospitalization); (c) symptoms/signs (fever, diarrhea, cough, chest and abdominal pain, anosmia, dysgeusia including day of onset, altered consciousness, and dyspnea); (d) previous comorbidities (smoking habit, diabetes, hypertension, chronic cardiac disease, CKD, CLD, chronic respiratory disease, and chronic neurological disorders or malignancies); (e) main COVID-19 administered drugs (cortison, monoclonal antibodies, and antivirals) at the time of hospital admission; and (f) main laboratory data (creatinine and eGFR calculated by the CKD-EPI equation). CKD was diagnosed according to the most recent guidelines, thus identifying five groups according to the stage of disease: eGFR < 15 mL/min/1.73 m^2^ (stage 5), 15–29 mL/min/1.73 m^2^ (stage 4), 30–59 mL/min/1.73 m^2^ (stage 3), 60–89 mL/min/1.73 m^2^ (stage 2), and >90 mL/min/1.73 m^2^ (stage 1) [14].

A diagnosis of diabetes was made according to the American Diabetes Association (ADA) and the most recent guidelines based on the clinical history of the patient and laboratory exams at admission [15]. A diagnosis of hypertension was made according to the most recent guidelines and the patient’s medical history [16]. Chronic cardiac diseases, which include heart failure, ischemic cardiopathy, previous acute myocardial infarction (AMI), atrial fibrillation. and valvulopathy, were instead diagnosed according to anamnestic and clinical data only. CKD, CLD, chronic respiratory diseases (asthma, lung fibrosis, and chronic obstructive pulmonary disease), and neurologic disorders and malignancies were diagnosed according to clinical history.

### 2.3. Statistical Analysis

Continuous variables were expressed as either mean data and standard deviations (SDs) or median data and interquartile ranges (IQRs) according to their distribution and results obtained from the Shapiro–Wilk test, while categorical data were expressed as numbers and percentages. Missing data were managed as follows: (a) for categorical data, missing information was managed by creating a specific ’Missing’ category for each variable under analysis; and (b) for continuous data, no imputation method, single or multiple, was used and variables with missing information were expressed in the table as not applicable (N/A). Population data were divided in different groups based on their eGFR. An overall *p*-value was used to express the difference between stages (either ANOVA or Kruskal–Wallis test for continuous data, depending on data distribution, and chi-squared or Fisher’s exact tests were used for dichotomous/categorical data, depending on the sample size) and a *p*-value was used for trends to look for a linear, increasing, or decreasing association trend (non-parametric test for a trend across ordered groups or a chi-square statistic for the trend). Univariable and multivariable Cox’s regression models were used to evaluate the associations between risk mortality and clinical variables (a univariable model is shown in the Supplementary Materials). The hazard ratio and 95% confidence intervals (HR-95% CI) were calculated for all models. Variables were included in the multivariable model according to the following rules: (a) significance at the univariable analysis (*p* < 0.05); (b) lack of co-linearity (also explored with preliminary analysis); (c) sex variable, although not significant, was added in the analysis; and (d) multivariable models were compared by applying the likelihood-ratio test (LR test) in the case of nested models or the Bayesian information criterion (BIC) in the case of non-nested models, and the preferred models had lower values. Based on the Cox’s regression model results, a survival analysis was performed to evaluate the in-hospital mortality risk related to the variable stages of eGFR, where a statistically significant *p*-value was considered by a log-rank test. A spline function for the continuous variable eGFR, a Kaplan–Meier survival analysis, and a Sankey plot were performed. All procedures were performed through the RStudio^®^ software (RStudio, Boston, MA, USA).

## 3. Results

### 3.1. Characteristics of the Study Population

A total of 1403 hospitalized patients with positive swabs for SARS-CoV-2 were considered eligible for the study, and of these, 157 were excluded due to the absence of data on admission and/or at discharge to calculate the survival analysis. Of the patients, 1246 included in the study were males (65.3%) with a mean age of 64 years (SD 14.6) and a median hospitalization duration of 15 days (IQR 9–22 days). The median time elapsed between the onset of symptoms and hospitalization was 6 days (IQR 3–9 days). At the time of hospitalization, 28.0% of the patients did not show any symptom of acute respiratory distress syndrome (ARDS), while moderate and severe symptoms were observed in 19.2% and 16.6% of the patients, respectively.

Twenty-nine patients (2.3%) showed moderate to severe impaired consciousness according to the Glasgow Coma Scales (GCS/15). Moreover, as for the respiratory severity scale (RSS), 596 patients (47.8%) received respiratory support at the time of hospitalization either with Venturi masks or nasal cannula, and 32.9% required either non-invasive ventilation (NIV) or orotracheal intubation (OTI). 

The study population was stratified according to the stages of eGFR (1, 2, 3, 4 and 5). *p*-values were not calculated for age, creatinine, and eGFR because these variables were already considered during the eGFR measurement. All clinical characteristics at admission are reported in Table 1.

### 3.2. Mortality Risk and Clinical Prognostic Factors

During the study period, 258 in-hospital mortality events were recorded, with a cumulative incidence of 20.7%. 

The univariate Cox’s regression analysis showed a significant association between mortality risk and age (HR 1.06, 95% CI 1.05–1.07; *p* < 0.001) and respiratory rate (HR 1.11, 95% CI 1.09–1.13; *p* < 0.001). A significant risk of mortality on a moderate ARDS scale (moderate vs. absent -ref-, HR 3.26, 95% CI 2.00–5.33; *p* < 0.001) and severe ARDS scale (severe vs. absent -ref-, HR 8.46, 95% CI 5.38–13.32; *p* < 0.001) was demonstrated. In addition, the respiratory severity scale revealed higher mortality rates for subjects treated with NIV (NIV vs. none -ref-, HR 1.36, 95% CI 2.19–6.97; *p* < 0.001) and OTI (OTI vs. none -ref-, HR 86.85, 95% CI 15.49–59.91; *p* < 0.001). A moderate-to-severe impaired consciousness evaluated through GCS/15 showed a worse clinical outcome (HR 8.03, 95% CI 5.22–12.35; *p* < 0.001). Several comorbidities present at the time of admission, including chronic cardiac disease (HR 2.65, 95% CI 2.06–3.41; *p* < 0.001), CKD (HR 3.33, 95% CI 2.47–4.49; *p* < 0.001), hypertension (HR 1.65, 95% CI 1.25–2.16; *p* < 0.001), diabetes (HR 1.73, 95% CI 1.33–2.26; *p* < 0.001), smoking (HR 1.70, 95% CI 1.15–2.49; *p* = 0.007), chronic respiratory disease (HR 2.27, 95% CI 1.72–3.0; *p* < 0.001), and chronic neurological disorder (HR 1.64, 95% CI 1.14–2.35; *p* = 0.008), were significantly associated with poor outcomes. Finally, the analysis showed a significant association between mortality risk and treatment with antivirals (HR 0.24, 95% CI 0.16–0.37; *p* < 0.001).

These results show a significant association between mortality risk and CKD, with more advanced stages of CKD increasing the likelihood of mortality (HR 5.66, 95% CI 3.79–8.43; *p* < 0.001 for stage 2, HR 9.82, 95% CI 6.01–16.04; *p* < 0.001 for stage 3, and HR 14.04, 95% CI 8.86–22.25; *p* < 0.001 for stage 4). These results are shown in Supplementary Materials Table S1. The multivariable model was defined according to the following concepts: (1) in the choice between ARDS scale and RSS, given the overlap of clinical nature, we chose the best univariable model evaluated with the BIC (Bayesian information criterion) score; (2) the variables CLD and malignancies were statistically significant in the previous paper, and thus we performed the likelihood-ratio test (LR test) to compare the quality of the model’s adaptation; (3) we omitted the variable ‘CKD’ at the expense of the variable ‘stages of CKD’, according to the Bayesian information criterion (BIC) score; and (4) the interaction between the eGFR stages and antivirals was tested, but with no significant results. Significance was partially confirmed by the Cox’s multivariable regression model, which also demonstrated how the risk of mortality was strongly associated with the severity of CKD (HR 2.94, 95% CI 1.76–4.91, *p* < 0.001 for stage 3; HR 3.68, 95% CI 1.90–7.12, *p* < 0.001 for stage 4; and HR 7.25, 95% CI 4.04–13.01, *p* < 0.001 for stage 5, respectively, against stage 1 as reference). The multivariable Cox’s regression model results are shown in Table 2.

**Figure 1:**
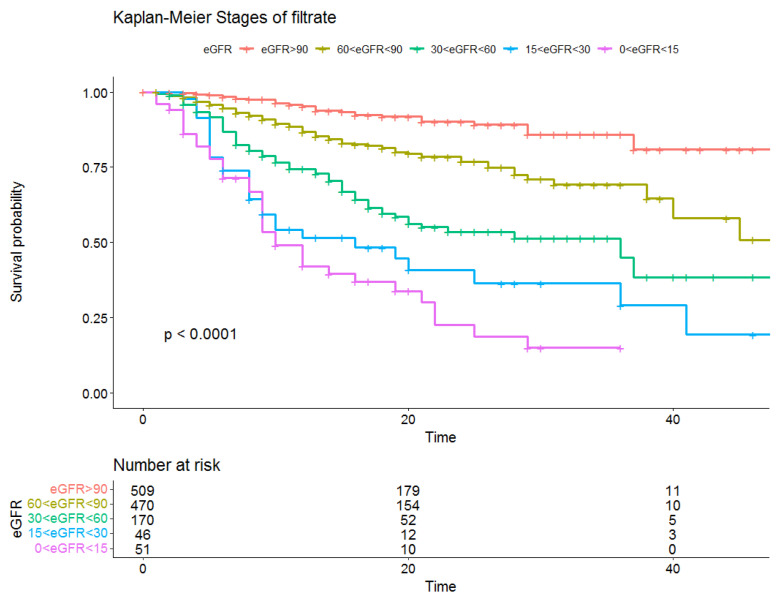
Kaplan–Meier survival analysis according to the stage of CKD, and the eGFR risk table.

**Figure 2:**
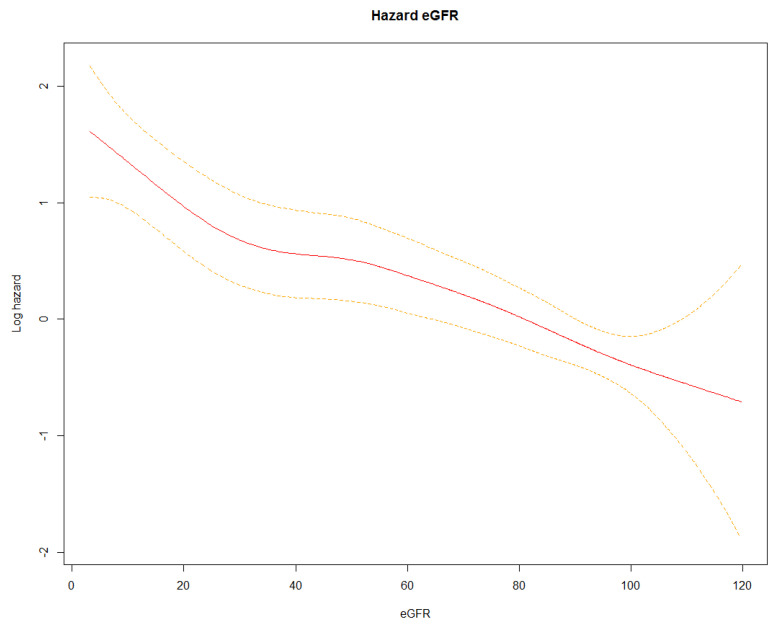
Spline function of the risk of mortality. Red line: Log hazard value; Dotted yellow lines: Confidence interval values.

We then conducted a survival analysis considering a hospitalization duration of 45 days as the maximum value. The resulting Kaplan–Meier described survival by showing a progressive and statistically significant difference (log-rank test *p* < 0.0001) in mortality according to the stage of eGFR, which increased with the decrease in eGFR (Figure 1). This result was also confirmed by the Spline function, which showed a mostly linear decrease in the risk of mortality as the eGFR increased (Figure 2). 

### 3.3. Evaluation of Admission/Discharged eGFR Stage 

Among the 1246 overall population patients, 1087 showed both eGFR and creatinine values on admission and on discharge. On admission, 450 patients displayed renal functions at stage 1 (41.4%), 409 at stage 2 (37.6%), 147 at stage 3 (13.5%), 38 at stage 4 (3.5%), and 43 at stage 5 (3.9%). At discharge, 506 patients displayed renal functions at stage 1 (46.5%), 358 (32.9%) at stage 2, 120 at stage 3 (11.0%), 48 at stage 4 (4.3%), and 55 at stage 4 (5.1%). 

At the end of hospitalization, of the 450 patients who had been at stage 1 on admission, 376 (83.6%) remained at stage 1 and 54 (12.0%) had worsened to stage 2, 6 (1.3%) to stage 3, 12 (2.7%) to stage 4, and 2 (0.4%) to stage 5. Of the 409 subjects who had been at stage 2 on admission, 115 (28.1%) had improved to stage 1 at the end of hospitalization, 234 (57.2%) remained at stage 2, 38 (9.3%) had deteriorated to stage 3, 10 (2.4%) had deteriorated to stage 4, and 12 (2.9%) had deteriorated to stage 5. From the subjects who were initially at stage 3 on admission (147), 11 (7.5%) had improved to stage 1 and 62 (42.2%) had improved to stage 2, 56 (38.1%) remained at stage 3, 13 (8.8%) had worsened to stage 4, and 5 (3.4%) had worsened to stage 5. From the patients who were at stage 4 on admission (38), 2 (5.3%) had improved to stage 1, 4 (10.5%) had improved to stage 2, 15 (39.5%) had improved to stage 3, 9 (23.7%) remained at stage 4, and 8 (21.1%) had deteriorated to stage 5. From the patients who were initially at stage 5 on admission (43), 2 (4.7%) had improved to stage 1, 4 (9.3%) had improved to stage 2, 5 (11.6%) had improved to stage 3, 4 (9.3%) had improved to stage 4, and 28 subjects remained at stage 5 (65.1%).

**Figure 3:**
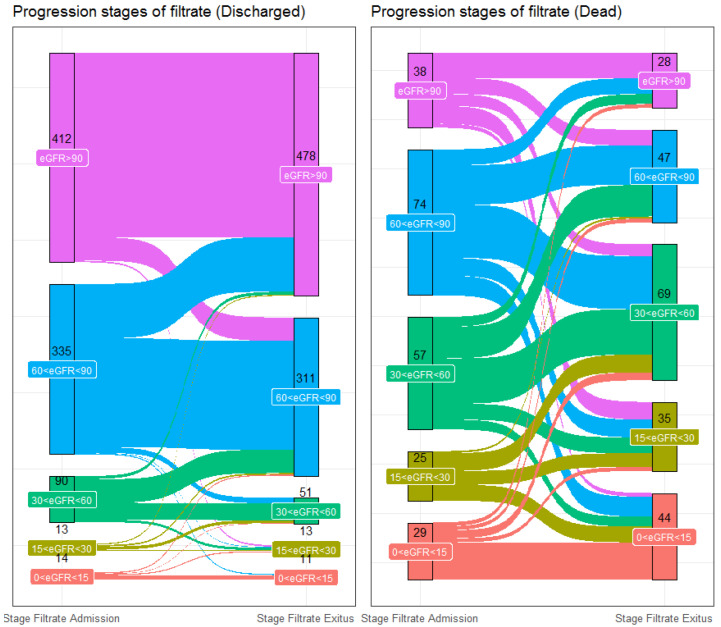
Sankey plot showing the progression of the stages of eGFR.

The variation across the stages of eGFR is described in the Sankey plot, which shows the change in the eGFR stages of the patients (discharged and dead) during hospitalization (Figure 3).

## 4. Discussion

The findings of this paper concern the so-called “second and third wave” of the Italian COVID-19 pandemic peak. The epidemiological data of that period showed an increased virus spread and mortality rate when compared to the “first wave” [1]. This evidence occurred despite the increased clinical and pharmacological experience and the start of the immunization program in December 2021 in Italy. Many risk factors have been associated with acute respiratory distress syndrome development and worsened clinical outcomes from COVID-19 [17,18,19]. As shown in previous papers, some comorbidities are closely related to intensive care admission and poor prognosis (e.g., hypertension, CLD, diabetes, and CKD) [20]. However, in contrast to the first COVOCA article, here, CLD and malignancies were not statistically associated with poor outcomes. Low GCS and OTI values were independently associated with higher in-hospital mortality. This result could be affected by the different sample size of the study and the different variants of coronavirus apparent during the period of enrollment [12]. Regarding respiratory support, due to the rapid transition from one form of support to another, NIV, continuous positive airway pressure (CPAP), and nasal high flow were all included in the same group (NIV). This group was not associated with a high risk of mortality, contrary to the previous study [12]. The experience acquired by the physicians during the prior treatment of COVID-19 patients could be responsible for this observation. In contrast, OTI was still significantly associated with a higher mortality risk. This result is coherent with what has been observed elsewhere, likely due to a selection bias of patients on OTI. OTI is only considered in the most severe cases of respiratory and other organ failures, and outcomes are already likely to be poor [21]. Moreover, in the previous phase of the COVOCA study, we observed that no drug classes used during the pandemic (antivirals, antibiotics, hydroxychloroquine, anticoagulants, and monoclonal antibodies) significantly modified in-hospital mortality, regardless of when therapy began, for both the overall and net value of those already receiving NIV/OTI at hospitalization [22]. Instead, in this analysis, only antivirals showed a significant association with in-hospital outcomes (Supplementary Materials Table S1). 

CKD affects approximately 4.4% of the Italian general population aged 45–74 years and it is recognized as one of the main risk factors for COVID-19 contagiousness (55.8% relative difference prevalence compared to the general population) [23,24]. We reported that CKD (an eGFR of <60 mL/min/1.73 m^2^) affects approximately 20% of hospital inpatients. We developed the hypothesis that the greater prevalence of CKD in our study population might be due to the type of sample collected. CKD in this and in previous studies is a categorical variable evaluated through medical clinical data. For this reason, we decided to perform the analysis on CKD based on the eGFR values and the consequent stages of disease in order to obtain an objective descriptive analysis.

We have reported that subjects with an eGFR of <30 mL/min/1.73 m^2^ were at greater risk of in-hospital mortality when compared to the other stages of the disease. This data is consistent with previous evidence reporting an increased mortality risk in patients affected by CKD and with an eGFR of <30 mL/min/1.73 m^2^ (HR 2.52) [25]. In addition, it seems that CKD has a greater impact on outcome than other well-known risk factors such as diabetes and chronic heart disease [25,26].

The survival analysis appears to reflect the difference in outcome according to renal function, with a significant decrease in survival particularly in the final stages of CKD (Figure 1). Moreover, mortality and reduced renal filtrate rates appear to already be significantly associated after 40 days of follow-up, while in the general population, this becomes clear only after years of follow-up (Figure 1) [27,28]. It therefore seems that the viral infection may not only be an epiphenomenon of the disease, but also a cofactor of worsening clinical status.

The pathophysiological mechanisms of the viral replication and molecular events leading to renal impairment in COVID-19 are not well understood. However, it seems that the virus could directly infect the kidneys through adhesion mediated by ACE-2 receptors and cause cell damage through both direct and indirect mechanisms [29,30,31]. ACE-2 receptors are mainly located in the apical border of the proximal tubule and, to a lesser degree, in the glomerular parietal cells/podocytes, thus suggesting tubular injury as the main consequence of COVID-19 infection [29,32]. Other authors, through renal histopathological analysis, assert that the damage may directly result from receptor activation and indirectly through the activation of inflammatory and immune systems which are, in turn, responsible for both the glomerular and tubular damage [29,33,34]. Microscopy analyses have, in fact, shown that the presence of the virus can be associated with a loss of brush border and nonisometric vacuolation, the tubulo-interstitial infiltration of macrophages, and the presence of complement factors (C5b-C9) [29,33,34]. Such damage would result in a worsening of renal function, acute disease through the manifestation of acute kidney injury (AKI), and a further worsening of pre-existing CKD. The tubular damage seems to be the reason for the worsening prognoses in patients with AKI and CKD compared to the rest of the population affected by COVID-19. As shown in Figure 1 and Figure 3, in our sample, some patients had a rapid worsening of renal function, and this result was closely linked to mortality. In a proportion of subjects, there was a decline in renal function from admission to discharge (Figure 3). This impairment could be strictly associated with the viral infection, as has been previously described in the literature [35,36,37].

Many authors have questioned which pharmacological approaches could prevent the renal function decline seen in these subjects. Some authors suggest that inhibition of the spike protein mediated by the vaccine, as well as furin, would be useful in preventing viral replication at renal sites. Both the inhibition of transmembrane protease serine 2 (TMPRSS2) and viral RNA replication by antiviral agents may be valid pharmacological alternatives [29,38]. Other authors have observed the involvement of the renin-angiotensin-aldosterone system (RAAS) and its complement in the pathogenesis of virus-induced kidney damage, thus hypothesizing that the pharmacological inhibition of RAAS and its complement may prevent kidney impairment. However, there is currently no clear evidence to support these hypotheses and discordant results have been shown in the literature. In our study, no pharmacological treatment conditioned the patient prognosis according to the stage of eGFR (Figure 1).

The present study has some limitations. First, there was an absence of laboratory data on urinary protein and sediment measurements, and its inclusion would have allowed us to clarify the phenotype of the renal damage. Secondly, there was an absence of data collection during hospitalization from admission to discharge, as required by the study design. This data collection would have highlighted those forms of AKI that manifested within 48 hours. Thirdly, there was an absence of body mass index (BMI) and related data, which did not allow us to include this as a variable in the analysis. Fourthly, there was an absence of creatinine values collected before hospital admission. These values would have clarified the status of the patients’ kidney function before the development of the infection. Fifthly, the timeframe of enrollment was previous to the wide spread of the vaccination campaign against SARS-CoV-2 in Italy, and thus we did not have complete data for adding this variable to the statistical analysis. Ultimately, the absence of biopsy data did not allow us to clarify what type of histological renal damage occurred in the COVID-19 patients. However, it would have been very difficult to perform further renal investigations at that time due to the emergency status caused by the pandemic and the characteristics of the patients.

## 5. Conclusions

This study confirms the association between CKD, stage of eGFR at admission, and mortality rate in patients hospitalized for COVID-19.

We have proposed that eGFR levels can be considered as a direct measurement to estimate mortality risk. This finding has a strong clinical implication as it can help clinicians to identify subjects with the highest risk of intra-hospital mortality, despite the short duration of follow-up.

The progression of the disease needs to be carefully monitored in these patients, especially because it can increase the severity of CKD. Further investigations, especially histopathological analyses, are necessary to further clarify the pathophysiological mechanisms that link COVID-19 to kidney damage.

## Figures and Tables

**Table 1:** Baseline characteristics of the study population presented as overall data and according to the stage of eGFR.

Parameter	Overall	1	2	3	4	5	*p*-Overall	*p*-Trend
	(*n* = 1246)	(*n* = 509)	(*n* = 470)	(*n* = 170)	(*n* = 46)	(*n* = 51)		
Age, mean (SD)	63.5 (14.6)	53.6 (12.2)	68.5 (11.7)	74.4 (10.7)	73.5 (14.9)	70.2 (12.8)		
Sex, *n* (%)							<0.001	<0.001
M	814 (65.3)	392 (77.0)	267 (56.8)	98 (57.6)	25 (54.3)	32 (62.7)		
F	432 (34.7)	117 (23.0)	203 (43.1)	72 (42.4)	21 (45.7)	19 (37.3)		
Duration of hospitalization, median (IQR)	15 [9–22]	15 [10–23]	15 [10–22]	14 [8–21]	9.5 [6.0–19.8]	9 [5–17]	<0.001	<0.001
Any positive in the family, *n* (%)	226 (31.7)	102 (32.5)	96 (36.2)	23 (26.1)	2 (10.0)	3 (11.1)	0.009	0.017
First positive in the family, *n* (%)	352 (68.3)	145 (66.2)	128 (64.3)	51 (78.5)	12 (80.0)	16 (76.2)	0.159	0.071
Days before hospitalization, median (IQR)	6 [3–9]	7 [4–10]	5 [2–8]	5 [2–10]	7 [2.8–11.5]	4.5 [1.3–10.3]	0.112	0.023
Body temp (°C), mean (SD)	36.7 (0.9)	36.8 (0.9)	36.7 (0.8)	36.7 (0.8)	36.7 (0.9)	36.6 (0.8)	0.016	0.001
Respiratory rate (apm), median (IQR)	20 [16–24]	20 [16–23]	20 [16–24]	20 [16–25]	21 [18–25]	20 [16–25]	0.054	0.011
Heart rate (bpm), mean (SD)	85.9 (15.2)	86.9 (13.9)	84.3 (14.4)	85.6 (17.6)	89.5 (19.1)	89.5 (20.0)	0.012	0.052
Blood pressure (mmHg), mean (SD)								
Systolic	132.9 (18.5)	131.5 (16.4)	134.9 (18.1)	132.3 (19.7)	128.9 (24.2)	135.9 (28.5)	0.020	0.135
Diastolic	77.7 (11.1)	78.7 (9.9)	78.3 (10.9)	74.4 (11.2)	74.3 (15.2)	76.3 (15.6)	<0.001	<0.001
Oxygen saturation %, median (IQR)	94 [91–96]	95 [92–97]	94 [90–96]	93 [89–96]	92 [86.3–95.0]	95 [91.3–97.0]	<0.001	<0.001
ARDS Scale, *n* (%)								
Absent	349 (28.0)	160 (31.4)	132 (28.1)	41 (24.1)	7 (15.2)	9 (17.7)	<0.001	<0.001
Mild	284 (22.8)	118 (23.2)	110 (23.4)	42 (24.7)	3 (6.5)	11 (21.6)		
Moderate	239 (19.2)	106 (20.8)	94 (20.0)	27 (15.9)	7 (15.2)	5 (9.8)		
Severe	207 (16.6)	65 (12.8)	74 (15.7)	36 (21.2)	16 (34.8)	16 (31.4)		
Missing	167 (13.4)	60 (11.8)	60 (12.8)	24 (14.1)	13 (28.2)	10 (19.6)		
GCS/15, *n* (%)	.		.	.	.	.	0.016	0.002
Mild impaired consciousness	1076 (86.4)	452 (88.8)	404 (86.0)	145 (85.3)	35 (76.1)	40 (78.4)		
Moderate/severe impaired	29 (2.3)	7 (1.4)	11 (2.3)	4 (2.3)	4 (8.7)	3 (5.9)		
consciousness								
Missing	141 (11.3)	50 (9.8)	55 (11.7)	21 (12.4)	7 (15.2)	8 (15.7)		
Respiratory Severity Scale, *n* (%)								
None	241 (19.3)	132 (25.9)	71 (15.1)	28 (16.5)	4 (8.7)	6 (11.8)	<0.001	<0.001
Mask/Glasses/Cannula	596 (47.8)	230 (45.2)	229 (48.7)	87 (51.2)	22 (47.8)	28 (54.8)		
NIV	385 (31.0)	142 (27.9)	62 (34.5)	47 (27.6)	18 (39.1)	16 (31.4)		
OTI	24 (1.9)	5 (1.0)	8 (1.7)	8 (4.7)	2 (4.4)	1 (2.0)		
Chronic Cardiac Disease, *n* (%)	303 (25.2)	62 (12.5)	114 (24.8)	78 (48.8)	29 (70.7)	20 (41.7)	<0.001	<0.001
CKD, *n* (%)	118 (10.0)	4 (0.8)	15 (3.3)	35 (22.6)	17 (44.7)	47 (92.2)	<0.001	<0.001
Hypertension, *n* (%)	698 (57.3)	202 (40.6)	299 (64.4)	126 (77.3)	31 (72.1)	40 (78.4)	<0.001	<0.001
Diabetes, *n* (%)	274 (22.7)	57 (11.5)	114 (24.8)	67 (41.9)	14 (35.0)	22 (44.0)	<0.001	<0.001
Smoking, *n* (%)	129 (14.2)	56 (14.3)	41 (12.0)	18 (15.7)	8 (28.6)	6 (18.2)	0.155	0.199
CLD, *n* (%)	66 (5.6)	18 (3.7)	31 (6.8)	11 (7.0)	3 (8.1)	3 (6.5)	0.182	0.062
Chronic Respiratory Disease, *n* (%)	189 (15.8)	47 (9.5)	86 (18.9)	37 (23.1)	9 (23.7)	10 (20.8)	<0.001	<0.001
Chronic Neurological Disorder, *n* (%)	110 (9.1)	27 (5.8)	49 (10.9)	27 (16.6)	3 (7.3)	4 (10.2)	<0.001	0.006
Malign, *n* (%)	111 (9.3)	36 (7.3)	42 (9.3)	25 (15.7)	5 (12.5)	3 (6.4)	0.026	0.071
Laboratory								
Creatinine, median (IQR)	0.9 [0.7–1.1]	0.7 [0.7–0.8]	0.9 [0.7–1.0]	1.3 [1.1–1.5]	2.4 [2.0–2.7]	6.3 [4.6–8.1]		
eGFR, median (IQR)	85.1 [64.5–97.7]	99.9 [95.7–106.4]	78.2 [70.9–84.9]	48.1 [38.2–55.0]	23.8 [20.7–27.3]	7.3 [5.9–10.7]		
Drugs								
Cortison, *n* (%)	1141 (92.8)	468 (92.5)	436 (93.6)	152 (92.1)	39 (90.7)	46 (92.0)	0.931	0.780
Monoclonal Abs, *n* (%)	46 (4.1)	17 (3.6)	16 (3.8)	9 (5.9)	4 (11.1)	-	0.076	0.478
Antivirals, *n* (%)	325 (28.4)	169 (35.4)	131 (30.5)	24 (15.4)	1 (2.8)	-	<0.001	<0.001

Abbreviations: M: male; F: female; Abs: antibodies; ARDS: acute respiratory distress syndrome; Body temp: body temperature; GCS: Glasgow coma score; RSS: respiratory severity scale; NIV: non-invasive ventilation; OTI: orotracheal intubation; CKD: chronic kidney disease; CLD: chronic liver disease; eGFR: estimated glomerular filtration rate; Malign: malignancies. Chronic cardiac diseases (ischemic cardiopathy, previous acute myocardial infarction (AMI), heart failure, valvulopathy, and atrial fibrillation); CKD (chronic renal failure, glomerulonephritis, and dialysis), chronic respiratory diseases (chronic obstructive pulmonary disease, asthma, and lung fibrosis), CLD (chronic hepatopathy from HCV and HBV, cirrhosis, and NAFLD).

**Table 2:** Multivariable Cox’s regression model.

Parameter	Multivariable Analysis			
	HR	95% CI		*p*
Age	1.03	1.01	1.04	<0.001
Sex				
M (ref)	1			
F	0.76	0.55	1.03	0.079
Respiratory rate	1.08	1.06	1.11	<0.001
Respiratory severity scale				
None (ref)	1			
Mask/glasses/cannula	1.97	0.98	3.95	0.058
NIV	2.01	0.98	4.10	0.056
OTI	13.33	5.82	30.53	<0.001
GCS/15, *n* (%)				
Mild impaired consciousness (ref)	1			
Moderate/severe impaired	4.03	2.24	7.24	<0.001
consciousness				
Missing	0.81	0.43	1.53	0.524
Chronic cardiac disease	1.39	1.00	1.92	0.047
Antivirals	0.43	0.27	0.68	<0.001
Stages of eGFR				
1 (ref)	1			
2	1.71	1.08	2.70	0.022
3	2.94	1.76	4.91	<0.001
4	3.68	1.90	7.12	<0.001
5	7.25	4.04	13.01	<0.001

Abbreviations: GCS: Glasgow coma score; NIV: non-invasive ventilation; OTI: orotracheal intubation; CKD: chronic kidney disease; CLD: chronic liver disease; eGFR: estimated glomerular filtration rate; Malign: malignancies. Chronic cardiac diseases (ischemic cardiopathy, previous acute myocardial infarction (AMI), heart failure, valvulopathy, and atrial fibrillation).

## Data Availability

Not applicable.

## Supplementary Materials

The following supporting information can be downloaded at: https://www.mdpi.com/article/10.3390/jcm11206121/s1. Table S1. Univariable Cox’s regression models.
