# Supplementary material for: Association between Renal Function at Admission and COVID-19 in-Hospital Mortality in Southern Italy: Findings from the Prospective Multicenter Italian COVOCA Study
Source: J Clin Med. 2022 Oct 17;11(20):6121. doi: 10.3390/jcm11206121 (PMC9604778; doi:10.3390/jcm11206121)
Supplement: Supplementary file 1 [file jcm-11-06121-s001.zip › jcm-1896205-supplementary.pdf]

Supplementary Materials

Table S1. Univariable Cox's regression models.

	Univariable Analysis			
**Parameter**	**HR**	**95% CI**		***p***
**Age**	1.06	1.05	1.07	<0.001
**Sex**				
M (ref)	1			
F	1.02	0.79	1.32	0.86
**Body temp**	0.96	0.83	1.12	0.62
**Respiratory rate**	1.11	1.09	1.14	<0.001
**Heart rate**	1.01	1.00	1.02	0.07
**Systolic BP**	1.00	0.99	1.00	0.25
**Diastolic BP**	0.97	0.96	0.99	<0.001
**Oxygen saturation**	0.94	0.92	0.95	<0.001
**ARDS Scale**				
Absent (ref)	1			
Mild	2.15	1.29	3.58	0.003
Moderate	3.26	2.00	5.33	<0.001
Severe	8.46	5.38	13.32	<0.001
Missing	4.05	2.42	6.77	<0.001
**GCS/15**				
Mild impaired consciousness (ref)	1			
Moderate/Severe impaired consciousness	8.03	5.22	12.35	<0.001
Missing	1.63	1.15	2.32	0.006
**Respiratory Severity Scale**				
None (ref)	1			
Mask/Glasses/Cannula	3.29	1.86	5.82	<0.001
NIV	3.90	2.19	6.97	<0.001
OTI	30.47	15.49	59.91	<0.001
**Chronic Cardiac Disease**	2.65	2.06	3.41	<0.001
**CKD**	3.33	2.47	4.49	<0.001
**Hypertension**	1.65	1.25	2.16	<0.001
**Diabetes**	1.73	1.33	2.26	<0.001
**Smoking**	1.70	1.15	2.49	0.007
**CLD**	0.96	0.54	1.71	0.89
**Chronic Respiratory Disease**	2.27	1.72	3.00	<0.001
**Chronic Neurological Disorder**	1.64	1.14	2.35	0.01
**Malignancies**	1.33	0.91	1.93	0.13
**Cortison**	1.13	0.63	2.02	0.67
**Monoclonal Abs**	1.11	0.62	2.00	0.72
**Antivirals**	0.24	0.16	0.37	<0.001
**Stages of filtrate**				
1 (ref)	1			
2	2.60	1.78	3.80	<0.001
3	5.66	3.79	8.43	<0.001
4	9.82	6.01	16.04	<0.001
5	14.04	8.86	22.25	<0.001

Abbreviations: M: Male; F: Female; Abs: Antibodies; ARDS: Acute Respiratory Distress Syndrome; Body temp: body temperature; GCS: Glasgow Coma Score; RSS: Respiratory Severity Scale; NIV: Non-invasive ventilation; OTI: Orotracheal Intubation; CKD: Chronic Kidney Disease; CLD: Chronic Liver Disease; eGFR: estimated glomerular filtration rate; Malign: Malignancies. **Chronic cardiac diseases** (ischemic cardiopathy, previous acute myocardial infarction (AMI), heart failure, valvulopathy and atrial fibrillation); **CKD** (chronic renal failure, glomerulonephritis and dialysis), **chronic respiratory diseases** (Chronic obstructive pulmonary disease, asthma, lung fibrosis), **CLD** (chronic hepatopathy from HCV and HBV, cirrhosis, NAFLD).
